# Frequency distribution of genes encoding aminoglycoside modifying enzymes in uropathogenic *E. coli* isolated from Iranian hospital

**DOI:** 10.1186/1756-0500-7-842

**Published:** 2014-11-25

**Authors:** Neda Soleimani, Mahdi Aganj, Liaqat Ali, Leili Shokoohizadeh, Türkân Sakinc

**Affiliations:** Department of Bacteriology, Faculty of Medical Sciences, Tarbiat Modares University, Po Box:14115–158, Tehran, Iran; Department of Microbiology, Faculty of Medical Sciences, student research committee, Mashhad University of medical sciences, Mashhad, Iran; Division of Infectious Diseases, Department of Internal Medicine II, University Hospital Freiburg, Freiburg, 79106 Germany; Faculty of Biology, Albert Ludwigs University of Freiburg, Schänzlestraße 1, Freiburg, 79104 Germany; Department of laboratory medical Sciences, Faculty of Para Medical Sciences, Ahvaz Jondishapour, University of medical sciences, Ahvaz, Iran

**Keywords:** UPEC, Aminoglycoside modifying enzymes and UTI

## Abstract

**Background:**

*Escherichia coli* is considered as the most common cause of urinary tract infection (UTI) and acquired multiple resistances to a wide range of antibiotics such as aminoglycosides. Enzymatic alteration of aminoglycosides (AMEs) by aminoglycoside- modifying enzymes is the main mechanism of resistance to these antibiotics in *E. coli.* The aim of this study was detection and investigation of frequency of genes encoding aminoglycoside modifying enzymes (aac(3)-IIa and ant(2′′)-Ia) in UPEC isolated from hospitalized patients in teaching hospital of Tehran, Iran.

**Findings:**

A total of 276 UPEC were obtained from Urine samples in a hospital from Tehran. Antibiotic susceptibility to aminoglycosides was determined by disk diffusion method according CLSI guidelines in UPEC isolates. MICs of target antibiotics were determined by agar dilution method. All isolates were screened for the presence of the AMEs genes using the PCR. The results of disk diffusion showed 21%, 24.6%, 23.18%, 3.62% and 6.15% of isolates were resistant to Gentamicin, Tobramycin, Kanamicin, Amikacin and Netilmicin respectively. The agar dilution’s results (MICs) were high, 66.19% for Gentamicin. The aac (3)-IIa and ant(2″)-Ia genes were detected in (78.87%) and 47.88% of isolates respectively.

**Conclusions:**

This study shows the high frequency of genes encoding (AMEs) aac(3)-IIa and ant(2”)-Ia genes and their relationship between different aminoglycoside resistance phenotypes.

## Background

The aminoglycosides are potent bactericidal agents that inhibit bacterial protein synthesis by binding to the 30S ribosomal subunit. They are often used in combination with either a b-lactam or a glycopeptide, especially in the treatment of *Escherichia coli* UTI, as these drugs act synergically [1, 2]. The application may be limited by the appearance of resistant strains in treatment. Various mechanisms are playing a role in the development of aminoglycoside resistance but the presence of aminoglycoside modifying enzymes is the most clinical and epidemiological importance [3, 4]. These enzymes are divided into three classes: aminoglycoside acetyltrans- ferases (AACs), aminoglycoside phosphotransferases (APHs) and aminoglycoside nucleotidyltransferases (ANTs) [5].

Urinary tract infection is one of the most common human infections, especially in young women and frequently influenced by sex and age and 20-30% of young women experienced this infection [6, 7]. Due to the importance of the resistance to aminoglycosides and the role of ant(2)-Ia, aac(3)II-a genes in mechanism, the main purpose of this study is the detection of resistance genes ant(2”)-Ia, aac(3)II-a in clinical isolates of aminoglycoside resistant *E. coli* isolated from urine of hospitalized patients in teaching hospital of Tehran, Iran, to know the prevalence and frequency of distribution of genes encoding aminoglycoside in Iranian population.

## Materials and methods

### Sample collection

A total of 276 clinical isolates of *E. coli* from urine specimens were randomly collected from Tehran Heart center. All isolates were then identified as *E.coli* using conventional microbiological tests. Informed written consent was obtained from the patients and the study was approved by the institutional ethics committee of Department of Bacteriology, Faculty of Medical Sciences, Tarbiat Modares University, Tehran. Pure stock cultures of all isolates were stored frozen at −80°C in tryptic soy broth, containing 15% glycerol.

### Antibiotic susceptibility testing

Antimicrobial susceptibility test for different *E.coli* isolates was performed against Gentamicin (10 μg), Tobramycin (10 μg), Kanamycin (30 μg), Amikacin (30 μg) and Nethelmicin 30 μg, (Mast, UK) by disc diffusion method. Sizes were interpreted using standard recommendations of CLSI [8]. As the Gentamicin is the most applicable antibiotics to treat the infections due to gram positive and gram negative bacteria in Iranian patients, thus, Gentamicin MIC values were detected and the results were interpreted according to the CLSI guidelines.

### DNA extraction and Polymerase Chain Reaction

Total DNAs were extracted from bacteria isolates using the extraction kit (Bioneer, korea). The DNA was then extracted following the manufacturer’s instructions and electrophoresed on 0.8% agarose gel stained with ethidium bromide and visualized by UV-transillumination and gel documentation (Biometra Germani). Two sets of specific oligonucleotide primers for (aac(3)-IIa and ant(2′′)-Ia) genes were used as listed in Table 1 (designated by primer 3 software). The PCR mixture was prepared in a final volume of 25 μl. The amplification mixture consisted of template DNA (2 μl), 0.1 μM of the respective primers, 2.5 μl of a 10-fold concentrate PCR buffer, 200 μM of deoxynucleotide triphosphates, 2.5 μM MgCl2, and 1.5 U of Taq DNA polymerase (Cinna Gene). A thermocycler (Mastercycler gradient; Eppendorf, Hamburg, Germany) was programmed with the following parameters: after an initial denaturation for 5 min at 95°C, 30 cycles of amplification were performed with denaturation at 95.8°C for 1 min, annealing at 62°C for 1 min, and DNA extension at 72°C for 1 min, followed by a final extension at 72°C for 10 min. Then, 5 to 10 μl of the PCR products was analyzed by electrophoresis on o.8-1% (w/v) TAE agarose gel (Fermentas UAB, Vilnius, Lithuania) containing 0.5 μl/μl of ethidium bromide. Stained amplicons were then viewed on a UV transilluminator at 260 nm, (BioDoc- Analyse; Biometra, Goettingen, Germany). *klebsiella pneumoniae* 23823 [possessing (aac(3)-IIa+)] and *E. coli* 85085 [possessing (ant(2″)-Ia+)] by Statens Serum Institute of Denmark served as positive controls and *E. coli* ATCC 25922 as negative control (Table 1).

**Table 1:** **Primer sequences for aminoglycoside resistance genes detection**

Primers	Sequence (5’-3’)	Length
Fragment (700 bp)		
ant(2′′)-Ia fw	TCCAGAACCTTGACCGAAC	19
ant(2′′)-Ia rev	GCAAGACCTCAACCTTTTCC	20
Fragment (740 bp)		
aac(3)-IIa fw	CGGAAGGCAATAACGGAG	18
aac(3)-IIa rev	TCGAACAGGTAGCACTGAG	19

## Findings

### Antimicrobial susceptibility

**Figure 1:**
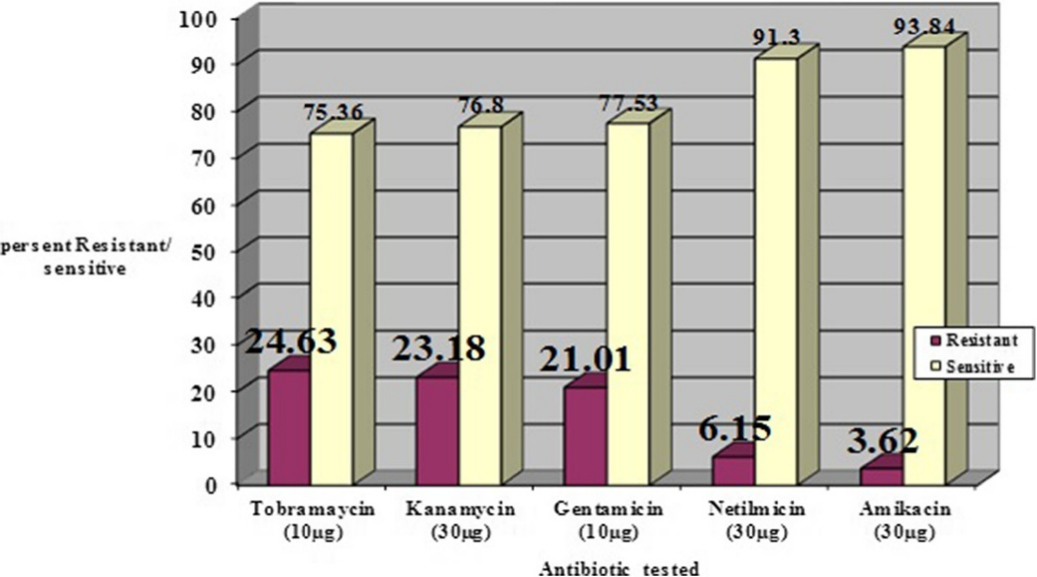
**Antibiotic sensitivity/resistance of** ***E. coli*** **strains isolated from the urine clinical specimens (n = 276).**

Antibiotic resistance analysis showed among 276 *E.coli* isolated from clinical specimens, simultaneous resistance to gentamicin, nethelmicin and kanamicin in 39% of isolates was the most common antibiotic resistance pattern among isolates under study (Table 2). The range of MIC for 5% Gentamicin was from 1 μg/ml to 512 μg/ml. MIC ≥ 64 were detected in more than 35% of isolates. Some isolates showed MIC of >512ug/ml. All antibiotic sensitivity/resistance of *E. coli* strains isolated from the urine clinical specimens are shown in Figure 1.

**Table 2:** **Antibiotic resistance patterns (%) of** ***E. coli*** **isolates**

Number of antibiotics resistant and showing pattern		Number of strains
Single antibiotic		3 (4.22%)
Two antibiotics	GM,TN (n = 4) or GM,K (n = 13)	17 (23.94%)
Three antibiotics	GM,TN, K	28 (39.43%)
Four antibiotic	GM,N,TN,K (n = 13) or GM,AK,TN,K (n = 6)	19 (26.76%)
Five antibiotics	GM,AK,N,TN,K	4 (5.63%)

GM, gentamicin; AK, amikacin; N, netilmicin; TN, tobramycin; K, kanamycin.

### Amplification and screening of genes encoding AME by PCR

**Figure 2:**
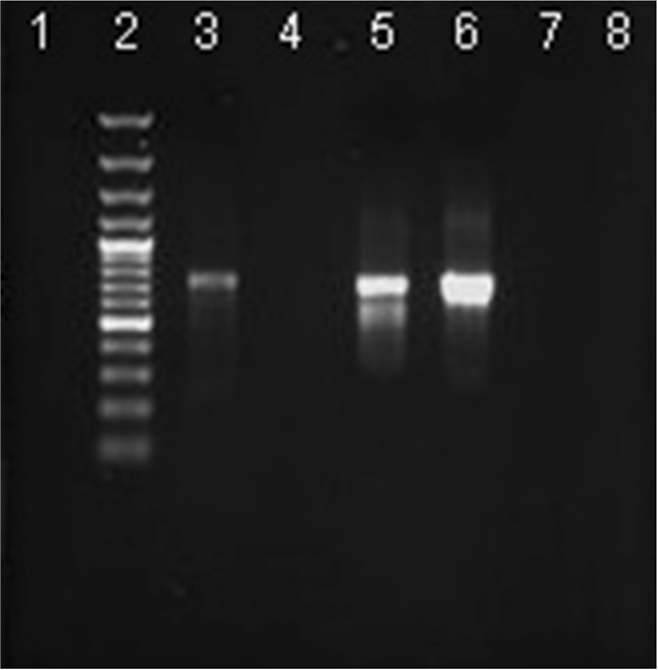
**Agarose gel electrophoresis of amplified DNA fragments by PCR from reference strains and clinical isolates of** ***E. coli*** **.** Lanes: 2, 100 bp Plus DNA ladder (GeneRuler_; Fermentas); 1, *E. coli* ATCC 25922 as negative control; 3, 23823 [aac(3)-IIa+] both were used as positive controls; 4–8, clinical isolates of *E. coli.*

**Figure 3:**
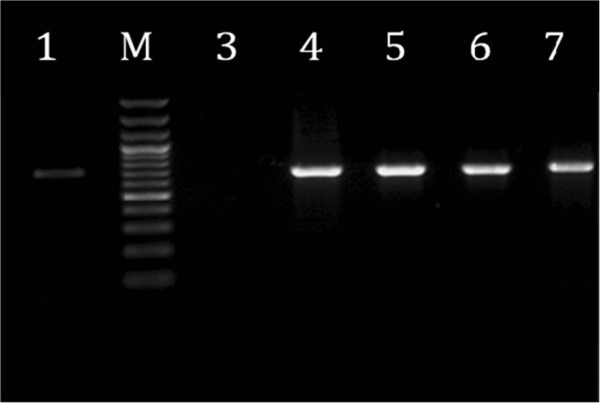
**Agarose gel electrophoresis of amplified DNA fragments by PCR from reference strains and clinical isolates of** ***E. coli*** **.** Lanes: M, 100 bp Plus DNA ladder (GeneRuler; Fermentas); 3, *E. coli* ATCC 25922 as negative control; 1, *E. coli* 85085 [(ant(2″)-Ia+)] both were used as positive controls; 4–7, clinical isolates of *E. coli.*

All isolates were screened for the presence of genes encoding the two AMEs, enzymes (AAC(3)-IIa and ANT(2′′)-Ia). Amplified DNA fragments of two different sizes (700 and 740 bp) were subjected to agarose gel electrophoresis and snapped by gel picture (Figures 2 and 3). Based on PCR results, The prevalence of aac(3)-IIa gene and ant(2”)-Ia gene were 47.88% and 78.87% respectively 32.39% of isolates only harbored the aac(3)-IIa and 7.04% ant(2”)-Ia. In addition our results, demonstrated the relationship between AME genes and different aminoglycoside resistance phenotypes (Table 3).

**Table 3:** **Relationship between AME genes and different resistance Aminoglycosid patterns**

***aac***(3)-II***a***	***ant***(2'')Ia	Resistant phenotype^*^
*+*	*+*	GM, AK, N, TN, K
*+*	*+*	GM, AK, N, TN, K
*+*	*+*	GM, N, TN, K
*+*	*_*	GM, N, TN, K
*+*	*+*	GM, TN
*_*	*+*	TN, K
*+*	*+*	GM, TN, K
*+*	*_*	TN
*_*	*+*	K
*_*	*_*	N
*+*	*_*	GM
*_*	*_*	AK

*GM, gentamicin; AK, amikacin; N, netilmicin; TN, tobramycin; K, kanamycin; +, present; −.

## Discussion

Besides the side effects and increasing resistance, aminoglycosides play an important role in curing bacterial infections. Modification of aminoglycosides by aminoglycosides modifying enzymes is the common resistance mechanism against aminiglycosides in *E.coli* as these enzymes are not capable of binding to ribosomes of the cell [9, 10]. Resistance against Gentamicin, Kanamycin, Cizomycin and Tobramycin is mediated by ANT(2”)-Ia enzyme which is coded by ant(2”)-Ia gene in *E.coli* and also simultaneous resistance to Gentamycin and Tobramycin, mediated by AAC(3)-IIa enzyme which is coded by aac(3)-IIa gene [11].

In this study the prevalence of ant(2”)-Ia,aac(3)-IIa resistance genes in 71 aminoglycosides resistant *E.coli* isolates among 276 UTI isolates was determined by PCR. It is implied that 24.63% of isolates were resistant to tobramycin and the resistance rate against other 130 antibiotics were as following; Kanamycin 23.18%, Gentamicin 21.01%, Netilmicin 6.15% and Amikasin 3.62%. In 1999 Van hoof R and his colleagues reported that among 897 blood 132 isolates of Entrobacteriacea, 5.9% of isolates were resistance against Gentamycin, whereas 7.7% of isolates were resistant against Tobramycin,7.5% against netilmicin and 8.2% against Amikacin [11, 12]. In 2006 Kong and 2010 Lang Hoo and colleagues reported: 44 clinicalisolates of *E.coli*, the resistance rate against aminiglycosides were: Amikasin 18.18%, Gentamicin 56.82% and Tobramycin 63.36% and among 249 clinical isolates of *E.coli* 83.83% were resistant to Gentamicin respectively [13, 14].

The respective studies suggested an increasing resistance against aminoglycosides but the contradiction in results is due to different geographical areas and various numbers of different isolates. PCR results showed that 78.87% of isolates contained aac(3)-IIa resistance gene. In 2004, Minard showed that 17% of animal and 33% of human isolates contained the aac(3)-IIa resistance gene, aph(3)-Ia was detected in 6.97% and 4% of human isolates of Kanamycin resistant. Also, *E.coli* in 8% of animal isolates and 7.04% of human isolates of neomycin resistant while ant(2”)-Ia gene was not detected in this study [7]. Jaconson et al. [15] studied 120 isolates of *E. coli* for occurrence of amino glycoside modifying enzymes namely ant(2”)-Ia, aac(3)-IIa and aac(3)-IV and also subjected the isolates to MIC for Gentamicin by dilution method. The *E.coli* isolates having aac(3)-IIa gene had high MIC’s, 32–512 mgs/ltr suggesting that, there is a correlation between MIC and specific ame production, but still not cleared [14]. Jacobson et al. also studied 76 isolates of Gentamicin resistant *E. coli* which are 63.15% and contained aac(3)-IIa gene, although, ant(2”)-Ia gene was not screened in this study [15]. In an epidemiological study in 2010 it was concluded that aac(3)-IIa (aaC2) gene was present in 84.1% of human isolates and 75.5% of animal isolates,while it was the common gene among the studied isolates [16].

Therefore, our results shows high frequency prevalence of aac(3)-IIa and ant(2”)-Ia genes, which were 47.88% and 78.87% respectively and also, demonstrated the relationship between AME genes and different aminoglycoside resistance phenotypes. According to the reviewed studies the prevalence of the respective genes has been increasing over time in various geographical patterns, which needs regular attention and determination.

## Conclusions

In conclusion, our data show high frequency distribution of aac(3)-IIa and ant(2”)-Ia genes and their relationship between AME genes and different aminoglycoside resistance phenotypes. Further experiments will be needed to clarify the exact mechanisms and functions of these genes to controlled high prevalence of urinary tract infections caused by EPEC strains, increasing resistance against antibiotics in order to select the best medicine to avoid this confrontation.
